# Editorial: Exposure to violence in children and youth during COVID-19 and mental health outcomes

**DOI:** 10.3389/frcha.2025.1675911

**Published:** 2025-09-02

**Authors:** Tracy Vaillancourt, Gary Slutkin

**Affiliations:** ^1^ University of Ottawa, Ottawa, ON, Canada; ^2^ Cure Violence Global, Chicago, IL, United States

**Keywords:** violence, children, adolescents, COVID-19, pandemic, mental health

**Editorial on the Research Topic**
Exposure to violence in children and youth during COVID-19 and mental health outcomes

According to the World Health Organization and secondary analyses, COVID-19 caused approximately 7 million confirmed deaths in just three years (2,020–2022), with excess-death estimates ranging from 14 to 18 million globally (1, 2). This total surpasses the combined five-year global death toll from tuberculosis (∼6 million), HIV/AIDS (∼3 million), and malaria (∼3 million) (3–5). In its wake, the pandemic has left behind a serious mental health crisis among youth, one that was already widespread but has now been further aggravated (6–8). This contagion continues to ripple outward, and we must address it.

Violence, like COVID-19, is an epidemic disease [see (9)]. It follows the patterns of all contagious processes: greater exposure increases risk, and the most vulnerable suffer the most severe consequences. Unlike traditional pathogens, however, violence spreads through observation, experience, and the witnessing of harm. Replication occurs in the brain via neural copying mechanisms in the cortex, reinforced by our need for approval and avoidance of disapproval, neurobiological processes shaped in the dopamine and pain systems. Trauma then acts as a powerful accelerant, increasing susceptibility to both perpetration and victimization.

Like other epidemics, violence breeds more violence. The invisible processes of exposure, vulnerability, and isolation create conditions for cyclical outbreaks. During times of social withdrawal, people develop heightened unmet social needs. When they re-emerge, exposure resumes, but this time with increased susceptibility. The COVID-19 pandemic, through its widespread fear, disconnection, and chronic stress, has amplified the conditions in which violence thrives.

Exposure to all forms of violence including child maltreatment, family violence, ethnic-based bullying, community violence, and self-harm, is a massive global health issue, particularly among children, youth, and young adults. The long-term effects extend across generations (10–12), with trauma and violence spreading not only through peer interactions and media, but also through broader societal and cultural channels. This process is known as *vertical transmission*. To date, most legal, educational, and social systems remain largely unequipped to recognize and interrupt this cycle.

Because pandemics disrupt societies far beyond illness and death, it is essential to examine how COVID-19 affected youth exposure to violence. The studies in this special section present global evidence that societal responses to manage the pandemic came at a cost disproportionately borne by young people, undermining their mental health, increasing their risk of future violence exposure, and compounding long-term health outcomes. Without urgent, coordinated interventions to interrupt these trajectories and strengthen systems of care, these harms are likely to persist.

## Family and household violence

McDowell et al. demonstrated that the likelihood of child maltreatment increased during the pandemic, despite some studies, especially those relying on administrative data, reporting decreases. The widespread shutdown or disruption of childcare centers, parenting programs, and clinical services left many children and youth more vulnerable to family violence. At the same time, families faced heightened stress from confinement, unemployment, and isolation. Crucial protective factors like peer relationships, school-based support, and community networks were stripped away, placing children and families at heightened risk.

McCarthy et al. provided further evidence that COVID-19 disproportionately harmed children with histories of maltreatment. These youth experienced more conflict with parents, siblings, and partners during the first three years of the pandemic compared to before. In families already struggling with violence, additional stress compounded risk, especially in the absence of intervention. Like McDowell et al., McCarthy et al. called attention to the failure of public health and support systems to respond proactively. The lesson is stark: any need left unmet becomes greater under stress.

Lee and Lee explored the intersection of family violence, violence against women, and the systemic dysfunction of U.S. family courts. In what they called a “deadly scourge,” they documented approximately 1,000 child deaths in custody-related contexts since 2008. These numbers rose nearly 50% during the COVID-19 years. They argued that family courts have too often enabled abuse, with many abusive fathers disproportionately winning custody. The consequences are devastating to children, but also for mothers, some of whom have been killed under these circumstances. Lee and Lee's work is a powerful indictment of how institutional failures perpetuate private violence.

## Peer-related violence and mental health

Vaillancourt and Brittain investigated peer victimization across early adulthood, showing that reduced peer contact during the pandemic was associated with decreased depressive symptoms among those previously victimized. In this natural experiment, social distancing acted as a protective factor, highlighting that separation from toxic peer environments can be a meaningful intervention. Their findings suggest that when victimization occurs, minimizing exposure can mitigate harm.

Farrell et al. examined whether the well-established link between bullying victimization and mental health difficulties weakened during the pandemic. Using a population-based sample of Canadian children and adolescents, they found that while bullying and mental health difficulties remained strongly correlated, the strength of the correlation was significantly weaker during the pandemic, especially for girls and adolescents. This was particularly true for verbal and social bullying, but not for physical or cyberbullying. The findings suggest that context, such as reduced in-person interaction, can attenuate the impact of victimization.

## Self-Harm and suicidal ideation

Xiao et al. studied suicidal ideation and attempts among adolescents and young adults in China during and after COVID-19 restrictions. Over 25% of respondents aged 12–24 reported having suicidal thoughts at least once in their lives, and more than 12% reported such thoughts in the previous 12 months. Risk was highest among middle school students and was strongly linked to depression, anxiety, and low social support. Additional risk factors included being female, attending boarding school, having divorced parents, *and prolonged quarantine*. Non-authoritative parenting also emerged as a significant predictor. These findings point to potential intervention targets and underscore the urgent need for sustained mental health support.

## Global and structural perspectives

Mbithi et al. investigated adolescent mental health in Kampala, Uganda, finding high levels of depression and anxiety, particularly among youth who were out of school during the pandemic. These adolescents were likely affected by compounded stressors: school closures, economic hardship, and family conflict. The study also documented increased household violence, highlighting the intersection of educational disruption and domestic strain. These findings illustrate how global inequalities exacerbate the effects of crises like COVID-19 and how places often overlooked in international discourse are sites of acute need and rising instability.

## Conclusion: a new way forward

Prior to the pandemic, over 30% of Canadian adults reported experiencing physical or sexual abuse during childhood (13). This level of maltreatment is not unique to Canada. Children worldwide are abused by their caregivers at alarming rates (14). And it's not just child abuse that is widespread. Bullying victimization, intimate partner violence, and community violence are also disturbingly common. This pervasive exposure to violence in childhood reflects a culture that has normalized harm. Although COVID-19 did not create these patterns, it has certainly intensified them.

The consequences of violence have never been confined to the domestic sphere or the neighbourhood. Around the world, trauma, fear, and isolation are also weaponized to erode democracy, foster authoritarianism, and inflame social division. These are not separate crises; they are syndromic expressions of the same underlying pathology: violence.

During the COVID-19 pandemic, public health efforts focused almost exclusively on reducing viral transmission, often at the expense of protecting children and adolescents from a parallel epidemic of violence. We overlooked the fact that, like COVID-19 and other viruses, violence spreads more readily when exposure increases, vulnerability deepens, and social structures break down. These are the conditions that were intensified by the pandemic. As a result, young people were not only at risk of contracting COVID-19; they were also at heightened risk of abuse, neglect, and psychological harm as protective systems collapsed, and stressors multiplied. We must not forget that vulnerability to violence and trauma rises during times of crisis, precisely when outreach, hotlines, and other forms of support are most urgently needed.
